# Long Lasting Modulation of Cortical Oscillations after Continuous Theta Burst Transcranial Magnetic Stimulation

**DOI:** 10.1371/journal.pone.0035080

**Published:** 2012-04-04

**Authors:** Nor Azila Noh, Giorgio Fuggetta, Paolo Manganotti, Antonio Fiaschi

**Affiliations:** 1 School of Psychology, College of Medicine, Biological Sciences and Psychology, University of Leicester, Leicester, United Kingdom; 2 Department of Basic Medical Sciences 1, Faculty of Medicine and Health Sciences, Universiti Sains Islam Malaysia (USIM), Level 13, Menara B, Persiaran MPAJ, Pandan Indah, Kuala Lumpur, Malaysia; 3 Department of Neurological, Neuropsychological, Morphological and Movement Sciences, Section of Clinical Neurology, G.B. Rossi Hospital, University of Verona, Verona, Italy; University of Bologna, Italy

## Abstract

Transcranial magnetic theta burst stimulation (TBS) differs from other high-frequency rTMS protocols because it induces plastic changes up to an hour despite lower stimulus intensity and shorter duration of stimulation. However, the effects of TBS on neuronal oscillations remain unclear. In this study, we used electroencephalography (EEG) to investigate changes of neuronal oscillations after continuous TBS (cTBS), the protocol that emulates long-term depression (LTD) form of synaptic plasticity. We randomly divided 26 healthy humans into two groups receiving either Active or Sham cTBS as control over the left primary motor cortex (M1). Post-cTBS aftereffects were assessed with behavioural measurements at rest using motor evoked potentials (MEPs) and at active state during the execution of a choice reaction time (RT) task in combination with continuous electrophysiological recordings. The cTBS-induced EEG oscillations were assessed using event-related power (ERPow), which reflected regional oscillatory activity of neural assemblies of θ (4–7.5 Hz), low α (8–9.5 Hz), µ (10–12.5 Hz), low β (13–19.5 Hz), and high β (20–30 Hz) brain rhythms. Results revealed 20-min suppression of MEPs and at least 30-min increase of ERPow modulation, suggesting that besides MEPs, EEG has the potential to provide an accurate cortical readout to assess cortical excitability and to investigate the interference of cortical oscillations in the human brain post-cTBS. We also observed a predominant modulation of β frequency band, supporting the hypothesis that cTBS acts more on cortical level. Theta oscillations were also modulated during rest implying the involvement of independent cortical theta generators over the motor network post cTBS. This work provided more insights into the underlying mechanisms of cTBS, providing a possible link between synchronised neural oscillations and LTD in humans.

## Introduction

The rise of research investigating human cerebral plasticity was partly due to repetitive transcranial magnetic stimulation (rTMS) that is able to non-invasively modulate cortical excitability but the after-effects of which are short lasting [1]. Transcranial magnetic theta burst stimulation (TBS) differs from other high-frequency rTMS protocols because it is able to extend the after-effects of the induced plastic changes up to an hour despite its lower stimulus intensity and shorter duration of stimulation [2], [3]. These prolong after-effects and its safety efficacy makes TBS popular in research exploring the therapeutic potential of non-invasive brain stimulation [4], [5] although the precise underlying neural mechanisms are still poorly understood [6]. On the synaptic level, long-lasting changes induced by TBS emulate the mechanisms of synaptic plasticity of long-term potentiation (LTP) and long-term depression (LTD) in the hippocampus [2], [3], [6]. However, what remain unclear are the effects of TBS on regional oscillatory activity of neural assemblies [7].

Although there are evidences that linked abnormal oscillatory activity with the neurological and psychiatric disorders [8]–[12] and the increasing use of TBS in clinical setting, studies investigating the modulatory effect of TBS on network oscillations are surprisingly scarce. A study looking at network oscillations post continuous TBS (cTBS) on the frontal eye field of four healthy subjects demonstrated a higher electroencephalography (EEG) synchronisation in all frequency bands in the stimulated cerebral hemisphere relative to the non-stimulated hemisphere up to one hour [7]. However, in their study, the authors used a modified theta burst paradigm (30 Hz bursts repeated at 6 Hz) with a higher stimulation intensity (80% resting motor threshold), making direct comparison with the original protocol [2] problematic. Another recent study investigating the effects on baseline EEG and motor learning after cTBS on the motor cortex showed that cTBS had no effect on the EEG power spectra [13]. The authors suggested that EEG is not useful to predict theta burst plasticity-like mechanisms, however, instead of using multichannel EEG, the power spectra was derived from a single electrode of C3. Therefore, in the present study, we addressed the lack of knowledge of cTBS effects on motor system oscillations and their correlation with behavioural measurements by applying the original cTBS protocol (cTBS 300 pulses at 50 Hz repeated every 5 Hz) in 26 healthy subjects.

Previous studies examining post-TBS effects mainly rely on muscular responses of motor evoked potentials (MEPs) to indirectly measure the cortical excitability [2], [14]–[17]. They revealed that continuous TBS (cTBS) suppressed MEPs amplitude for 20 to 60 minutes and the longer-lasting plasticity effect emulated the mechanism of LTD. It is important to note that LTP and LTD–the mechanisms involved in synaptic plasticity–are monosynaptic events of chemical synaptic transmission demonstrated at the glutamatergic synapses of the hippocampus. However, MEPs that are commonly used in TBS experiments as index of cortical excitability and LTP/LTD events are polysynaptic measurement, separated by at least three synapses from the TMS source (synapses onto corticospinal neurons; synapses onto motor neurons of the spinal cord; and the neuromuscular synapses)[18], [19]. This study moves beyond MEPs and proposes another sensitive measure, the EEG signals to find evidence of plasticity-like mechanism induced by theta burst stimulation. EEG oscillatory waves are derived from the brain’s electrical activity through the synchronous excitatory and inhibitory input of the cortical pyramidal dendrites [20]–[22]. Due to its direct measurement of cortical excitability [23] and its closer proximity to the TMS source [18], EEG can be another sensitive technique besides MEPs to provide accurate cortical readout after magnetic stimulation. In the present study, we assessed the cortical excitability after the application of cTBS protocol using both EEG and MEPs.

The first goal of this study was to compare the temporal dynamics of human cortical excitability post-cTBS using both behavioural and electrophysiological measurements. Our second aim was to investigate how preconditioning motor cortex with high-frequency cTBS affect the subsequent patterns of oscillatory brain rhythms. Therefore, we delivered sub-threshold high-frequency cTBS over the left primary motor cortex (M1) and measured the cortical readouts via behavioural measurements of MEPs and a motor choice reaction times (RT) task, while simultaneously recording the electrophysiological measurements of EEG oscillatory activities at both rest and active states. Evaluation of EEG oscillatory phenomenon to cTBS was quantified by spectral analysis; frequency ranges of θ (4.0–7.5 Hz), low α (8.0–9.5 Hz), µ (10.0–12.5 Hz), low β (13.0–19.5 Hz), and high β (20.0–30.0 Hz) were chosen for analysis. The results showed the different temporal dynamics between EEG and MEPs with longer-lasting increase of EEG cortical oscillations relative to MEPs amplitude suppression post-cTBS. Overall we showed that cTBS interfered with motor system oscillations, thus providing a possible link between synchronised cortical oscillations and LTD in humans.

**Figure 1:**
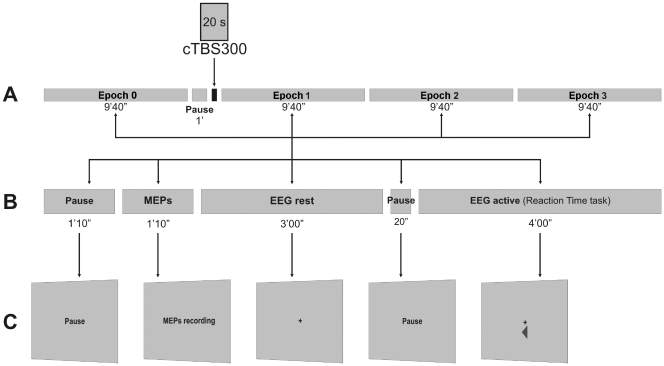
Experimental design: each subject underwent a 40-min recording session consisting of four blocks of 9′40″ duration each. Block 0 (baseline) preceded the application of cTBS 300 pulses over left M1; the remaining three blocks followed the cTBS (label A). Each block comprised of five events: 1) a pause of 1′10″, 2) MEPs recording for 1′10″, 3) EEG recording at rest of 3′00″, 4) a brief pause of 20″, and 5) EEG recording at active state, during the execution of a Reaction Time (RT) task, of 4′00″ duration (label B). Label C illustrated the example of the display presented on the computer screen during the experiment.

## Methods

### Participants

Twenty-six healthy volunteers (13 females; mean age, 26.7 years + 5.8 years) with no history of neurological disorder were randomly divided into two groups receiving either Active magnetic stimulation or Sham as control. Subjects were right-handed as assessed by the Edinburgh handedness inventory [24], and gave written informed consent prior to participation. The Local Ethical Committee of the G.B. Rossi Hospital “Borgo Roma” Verona, Italy, approved the entire experimental procedure.

### Experimental Design

Subjects were tested in a quiet dimly light room. They were seated in a comfortable armchair with eyes open, facing a computer screen. Each subject underwent a 40-min recording session consisting of four blocks of 9′40″ duration each. Block 0 (i.e. baseline) preceded the application of cTBS; the remaining three blocks followed the cTBS. Each block comprised of five events: 1) a pause of 1′10″, 2) MEPs recording for 1′10″, 3) EEG recording at rest for 3′00″, where a stationary black fixation cross symbol (0.8° of visual angle) on a grey background was presented at the centre of the screen, 4) a brief pause of 20″, and 5) EEG recording during the execution of a choice RT task of 4′00″ duration. Figure 1 shows the experimental paradigm.

### TMS and MEP

TMS was delivered through a figure-of-eight shaped coil (70 mm standard coil, Magstim Co., Whitland, Dyfed, UK) connected to a Magstim Super Rapid stimulator (Magstim, Whitland, Dyfed, UK). The coil was oriented so the induced electric current flowed in a posterior-anterior direction over M1. It was placed tangentially to the scalp with the handle pointing backwards and laterally at a 45° angle away from the midline, perpendicular to the central sulcus.

Electromyography (EMG) was recorded with Ag/AgCl surface electrodes placed over the right thenar eminence muscle (TE) with a belly-tendon montage. The amplified, bandpass-filtered (50 Hz to 5 KHz) EMG signal was fed into a Basic EMG Machine (Esaote Bio-medica, Florence, Italy). The optimal coil position was determined by moving it in 0.5 cm steps around the motor hand area of the left motor cortex where magnetic stimulation produced the largest MEP from the contralateral TE during relaxation (the “motor hot-spot”). Stimulus intensities were expressed as a percentage of the subject’s resting motor threshold (RMT); the minimum intensity over the motor hot-spot that could elicit an MEP of at least 50 µv in 50% of trials. The active motor threshold (AMT) was the minimum single pulse intensity with an MEP greater than 200 µV in more than 50% trials from the contralateral TE, during a sustained voluntary contraction of 20% maximum strength using visual feedback (i.e. dynamometer).

The patterns of cTBS consisted of a 20 s train of uninterrupted TBS with bursts of 3 pulses at 50 Hz repeated every 200 ms (i.e. 5 Hz) for a total of 300 pulses. cTBS were applied over left M1 and the stimulus intensity was at 80% of individual AMT.

### EEG Recording

Continuous EEG was recorded with a MR compatible EEG amplifier (SD MRI 32, Micromed, Treviso, Italy). Electrode montage and placement was according to the 10/10 system [25]. The EEG was continuously recorded from 30 Ag/AgCl electrodes sites (Fp1, AF3, AF4, F7, F3, Fz, F4, F8, FC5, FC1, FC2, FC6, T3, C3, Cz, C4, T4, CP5, CP1, CP2, CP6, T5, P3, Pz, P4, T6, PO3, PO4, O1, O2). According to the 10/10 system, the reference electrode was at AFz site, whereas the ground electrode was at FCz site as in previous studies using the same system [26]–[28]. The impedance was kept below 10 kΩ. The activities in the right TE muscle and in the right eye vertical electroculogram (vEOG) were bipolarly registered from two surface electrodes in two EMG channels. To ensure the subjects’ safety, the wires were carefully arranged to avoid loops and physical contact with the subject. To avoid electrical saturation of EEG channels induced by TMS, the EEG amplifier had a resolution of 22 bits with a range of ± 25.6 mV. An anti-aliasing hardware band-pass filter was applied with a bandwidth between 0.15 and 269.5 Hz. EEG data were sampled at a frequency of 1024 Hz using the software package SystemPlus (Micromed, Treviso, Italy).

### Reaction Time (RT) Task

On each trial, in the centre of the screen, a target stimulus was displayed of an arrowhead pointing to the left or right. The colour of the central arrow was isoluminant, either cyan or magenta. Subjects were asked to press the response key on the same side of the arrowhead (compatible condition) when the cyan arrow appeared on the screen. If magenta appeared, a response against the target arrow’s direction was required (incompatible condition). Two keyboard keys were used for response execution; the “C” key operated by the left index finger and the “M” key by the right index finger. Subjects were asked to respond as quickly and as accurately as possible. The visual display included a white fixation cross continuously present throughout the experimental blocks (0.5° of visual angle), whereas the arrowhead (1.5° of visual angle) was presented for 300 ms. Subjects were given 1500 ms to respond. Visual feedback, with duration of 300 ms, was then provided indicating whether subjects had executed an appropriate response. The time interval between successive trials was randomised between 2100 and 3100 ms (mean 2600 ms + 343 ms). In each block there were a total of 96 trials. Half of the trials required a “compatible” response (cyan arrowhead) and half required an “incompatible” response (magenta arrowhead). Subjects were given a practice block of 24 trials to become familiar with the task. The duration of the RT was 4′00″ in each of the four blocks. Correct responses were divided from errors and subjected to an absolute filtering criterion to remove anticipatory or overly delayed responses (RT < 150 ms and RT > 1300 ms).

### Data Analysis

Data were analysed using SPSS for Windows version 18.0. Repeated measures analyses of variances (ANOVAs) were used to compare variables before and after cTBS. Detail ANOVAs are in the respective analysis section of MEPs, RT and EEG. For each ANOVA, sphericity assumption was assessed with Mauchly’s test. Greenhouse-Geisser epsilon adjustments for non-sphericity were applied where appropriate. Significant results were subjected to post-hoc paired t-test adjusted for multiple comparisons with Bonferroni correction. For all statistical tests, *p* < .05 was considered significant.

### EEG Analysis

To demonstrate the cTBS-induced oscillations, EEG data were analysed with commercial software (Vision Analyser, Brain Vision, Munich, Germany). We analysed EEG signals for each block–block 0, block 1, block 2, block 3–and condition–EEG rest, EEG active (during a motor RT task)–for the two groups of Active cTBS and Sham cTBS. EEG at rest consisted of reference block (block 0) from 8′20″ to 5′20″ before cTBS and the three blocks after cTBS (block 1 from 2′20″ to 5′20″, block 2 from 12′00″ to 15′00″, and block 3 from 21′40″ to 24′40″). EEG active comprised of reference block (block 0) from 5′00″ to 1′00″ before cTBS and 5′40″ to 9′40″, 15′20″ to 19′20″, and 25′00″ to 29′00″ for the three blocks after cTBS.

EEG data were filtered (0.1–50 Hz, slope 24 dB/octave), and EMG signals were bandpass-filtered (30–300 Hz, slope 48 dB/octave). The following channels were selected for inspection: F3, Fz, F4, C3, Cz, C4, P3, Pz, and P4. A notch filter (50 Hz) was applied to all channels. After segmentation procedure into EEG segments of 2 seconds (2048 data points), a semi-automatic segment inspection-rejection procedure was applied to avoid, muscle or EOG activity. Epochs with eye movements and muscle or movement artefacts (as indicated by activity at electrodes exceeding ± 70 µV) were excluded from analysis. Overall, the number of accepted segments for each block at rest and active states ranged between 47 and 81 (94–164 seconds). To clarify whether there was a significant difference in the number of accepted segments between the different conditions of rest and active for the two experimental groups, we performed a repeated measures ANOVA with three within-subject factors: *block* (four levels – block 0, 1, 2 and 3), *condition* (two levels –rest and active)–and a between-subject factor of *group* (two levels – Active cTBS and Sham cTBS). The ANOVA for the number of accepted segments at rest or active state did not show any significant main effects or interactions.

For each subject, a discrete Fast Fourier Transform (FFT) of segments of 2048 data points (2 seconds) each was computed for all electrodes and then averaged under the same conditions. Power spectra were estimated for all frequency bins between 0.5 and 40 Hz (0.5 Hz of maximum bin width). Recordings were non-overlapping Hamming-windowed to control spectral leakage. The mean band power changes were then obtained by averaging the power values for θ (4–7.5 Hz), low α (8–9.5 Hz), µ (10–12.5 Hz), low β (13–19.5 Hz), and high β (20–30 Hz) frequency ranges chosen for analysis. In this study, we did not analyse the frequency band γ (>30 Hz) because the small amplitude of γ may overlap with the electrical muscle activity due to the similarity in their frequency characteristics [29].

In order to reduce the effects of inter-subject and inter-electrode variation in absolute spectral power values, circumvent the problem of a residual pre-existing difference between groups, and determine whether the expected differences in modulation of power between the two groups were caused by the experimental manipulation, we quantified the event-related relative changes of EEG power (ERPow) as in previous studies of TMS-EEG co-registration [26], [28], [30], [31]. Thus we calculated for each participant the percentage of increasing and decreasing of EEG oscillations post cTBS, with respect to the participant’s cortical oscillatory activity in the baseline (Block 0) for both EEG at rest and active conditions. An accepted event-related desynchronisation/synchronisation (ERD/ERS) procedure was used to quantify the event-related changes of EEG power at an electrode *x* (ERPow*_x_*) according to the equation (1).

ERPow*_x_*  =  [(Pow*_x event_* - Pow*_x reference_*)/Pow*_x reference_*] X 100

The ERPow (or ERD/ERS) transformation is defined as the percentage decrease/increase of instant power density at the *event* compared to a *pre-event* baseline. ERPow represents the TMS effects on regional oscillatory activity of neural assemblies. Therefore, ERPow increases imply synchronisation of the underlying neuronal populations and are expressed as positive values, while ERPow decreases are expressed as negative values [32]. ERPow were computed for four blocks of EEG at rest and active. Repeated measure ANOVAs were performed for both EEG at rest and EEG at active state for each frequency band of θ (4–7.5 Hz), low α (8–9.5 Hz), µ (10–12.5 Hz), low β (13–19.5 Hz), and high β (20–30 Hz), respectively. Each ANOVA had three within-subject factors–*block* (three levels – block 1, 2 and 3), *electrode* (nine levels – F3, Fz, F4, C3, Cz, C4, P3, Pz and P4)–and a between-subject factor of *group* (two levels – Active cTBS and Sham cTBS).

### MEP Analysis

The MEP amplitudes were measured in the resting right TE muscle at a stimulus intensity of 120% of the motor threshold. A total of ten TMS pulses were delivered in 1′10″ in each of the four blocks of the entire experimental session. The MEPs were measured at block 0 (baseline) from 9′30″ to 8′20″ before cTBS, block 1 from 1′10″ to 2′20″, block 2 from 10′50″ to 12′00″, and block 3 from 20′30″ to 21′40″ after cTBS. Statistical analyses were performed on normalised values of MEPs amplitudes and latency for both Active cTBS and Sham cTBS. The normalisation was with the MEPs values recorded at block 0, “pre-event” baseline. The peak-to-peak mean amplitudes or latency of MEPs were submitted to a repeated measure ANOVA with a within-subject factor *block* (three levels – block 1, 2 and 3) and a between-subject factor *group* (two levels - Active cTBS and Sham cTBS). In order to determine the correlation between the modulation of MEPs and the oscillatory indices after cTBS, a Pearson’s correlation (*p*<.05; two-tailed) coefficient was calculated between the changes of MEPs amplitude and ERPow modulation for the three blocks of time (block 1, 2 and 3) in all frequency bands over C3 electrodes as in previous studies [30], [31]. C3 was selected due to its location over M1.

### RT Analysis

The RT task performance was measured at block 0 (baseline) from 6′40″ to 1′00″ before cTBS, block 1 from 5′40″ to 9′40″, block 2 from 15′20″ to 19′20″, and block 3 from 25′00″ to 29′00″ after cTBS. Statistical analysis on the mean normalised correct trial scores (accuracy) and the mean normalised motor response onset latencies (RTs) were performed using repeated measure ANOVA with four within-subjects factors: *block* (three levels - 1, 2 and 3); *direction of the arrowhead* (two levels - left and right); *response position* (two levels - left and right); *colour of the arrowhead* (two levels – cyan and magenta); and a between-subject factor: *group* (two levels – Active cTBS and Sham cTBS). The normalisation of mean RTs and accuracy were with the behavioural performance recorded at block 0, a *pre-event* baseline.

## Results

### ERPow in the θ Band

**Figure 2:**
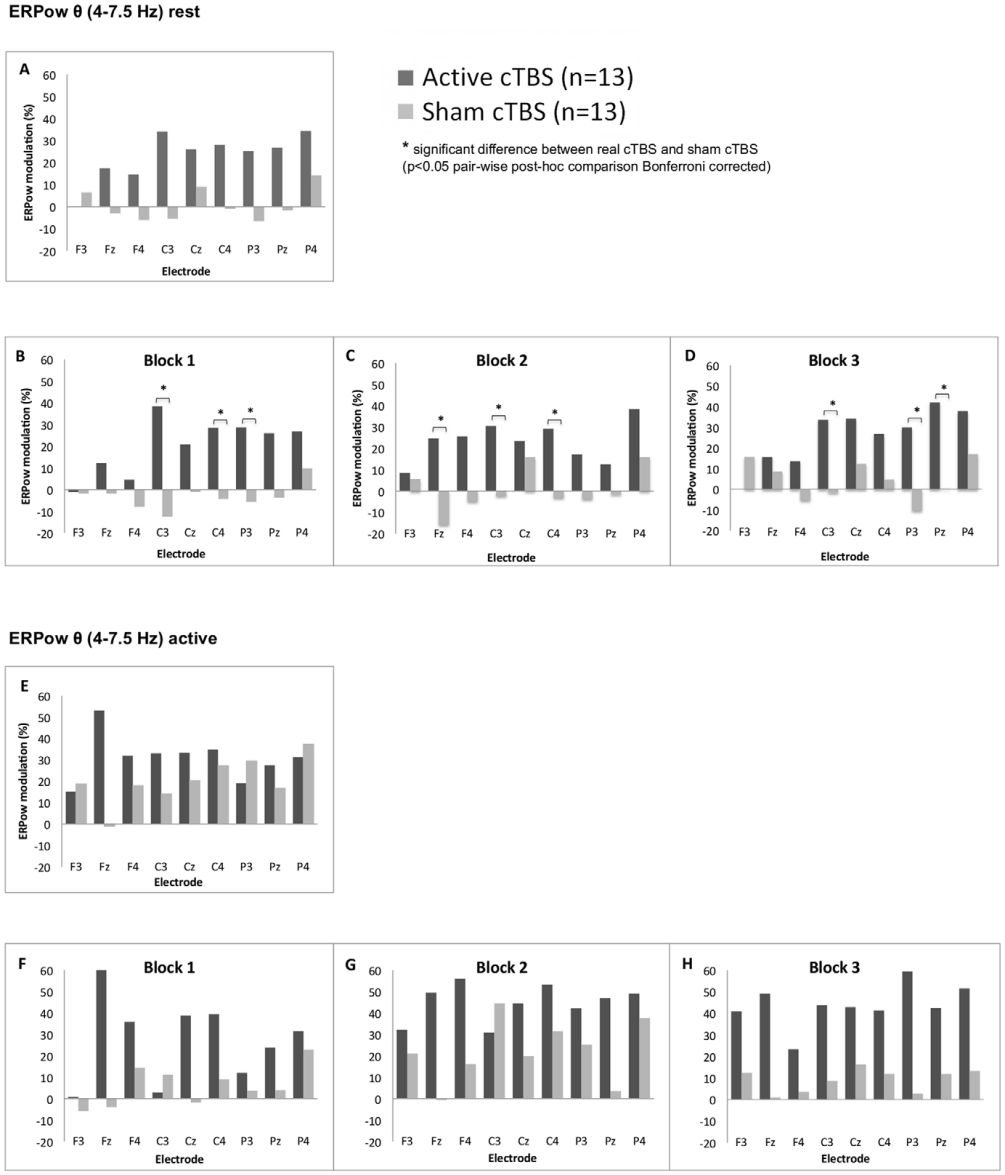
Grand average of ERPow transformation for θ (4–7.5 Hz) range. Post-cTBS after-effects are represented at rest and at active state as a function of the factors: group (Active cTBS and Sham cTBS), electrodes (F3, Fz, F4, C3, Cz, C4, P3, Pz, P4) and block of time (three levels – block one, two and three). The overall 30 minutes of post-cTBS after-effects on modulation of cortical oscillations for both groups of participants are shown at rest (label A) and active state (label E). Whereas the cTBS after-effects on ERPow separate for Block 1, Block 2 and Block 3 post stimulation are shown at rest (label B, C, and D) and at active state (label F, G, and H). ERPow at rest showed increased θ oscillations for Active cTBS group compared with Sham cTBS group across the three blocks post stimulation (See B–D).

The ANOVA of ERPow θ showed no significant main effects either at rest [*Block*: *F*_(1.6,38.3)_ = 0.92, *p* = .39, *η_p_^2^* = .04; *Electrode F*_(4.8,116.9)_ = 1.88, *p* = .11, *η_p_^2^* = .07] or during active condition [*Block*: *F*_(2,48)_ = 1.15, *p* = .16, *η_p_^2^* = .02; *Electrode F*_(8,192)_ = 1.31, *p* = .24, *η_p_^2^* = .05]. A significant interaction *Block* x *Electrode* x *Group* [*F*_(16,384)_ = 1.81, *p* < .05, *η_p_^2^* = .07] at rest demonstrated a significant increase in cortical oscillations for Active cTBS compared with Sham cTBS across the three blocks [block 1 for electrode C3 (38.4 vs. −12.5%), C4 (28.5 vs. −4.3%) and P3 (28.7 vs. −5.6%); block 2 in electrode Fz (24.6 vs. −15.7%), C3 (30.5 vs. −2.2%), C4 (29.2 vs. −3.1%); block 3 for electrode C3 (33.6 vs. −1.6%), P3 (29.9 vs. −10.3%) and Pz (42.0 vs. 0.3%)] (Fig.2B–D).

### ERPow in the Low α Band

The statistical analysis performed for ERPow low α at rest or active did not show any significant main effects or interactions.

### ERPow in the µ Band

**Figure 3:**
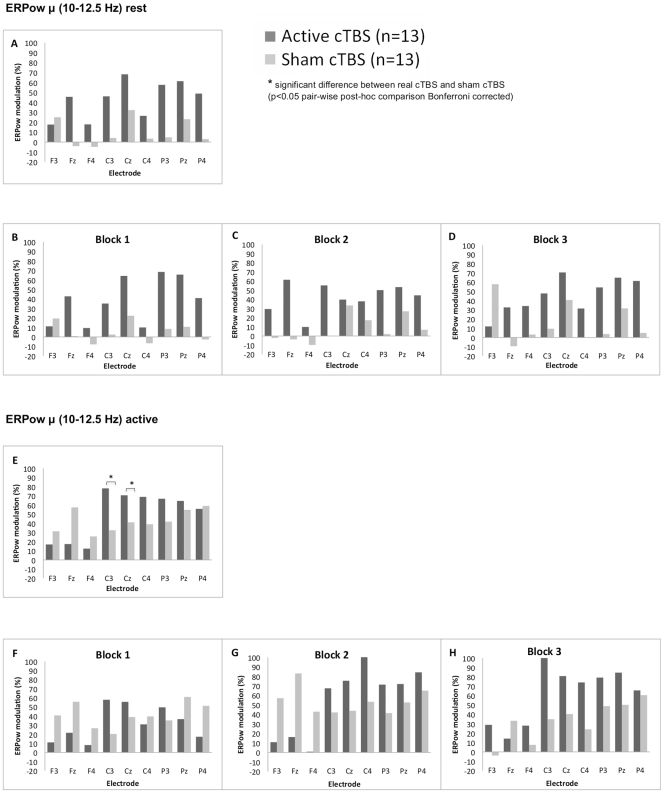
Grand average of ERPow transformation for µ (10–12.5 Hz) range. Post-cTBS after-effects are represented at rest and at active state as a function of the factors: group (Active cTBS and Sham cTBS), electrodes (F3, Fz, F4, C3, Cz, C4, P3, Pz, P4) and block of time (three levels – block one, two and three). The overall 30 minutes of post-cTBS after-effects on modulation of cortical oscillations for both groups of participants are shown at rest (label A) and active state (label E). Whereas the cTBS after-effects on ERPow separate for Block 1, Block 2 and Block 3 post stimulation are shown at rest (label B, C, and D) and at active state (label F, G, and H). ERPow at active state, during the execution of a motor task, showed higher EEG synchronisation for Active cTBS group compared with Sham cTBS group for C3 and Cz electrodes (See E).

The statistical analysis for ERPow µ at rest did not show significant effect *Block*: *F*_(2,48)_ = 1.53, *p* = .23, *η_p_^2^* = .06, but had significant effect *Electrode: F*_(4.9,116.7)_ = 3.16, *p* <.05, *η_p_^2^* = .12]. No interactions were significant. In the active state, the ANOVAs showed the following statistically significant main effects and interactions: *Electrode* [*F*_(4.6,111.1)_ = 4.75, *p* < .01, *η_p_^2^* = .17]; *Block* x *Group* [*F*_(2,48)_ = 3.60, *p* < .05, *η_p_^2^* = .13]; *Electrode* x *Group* [*F*_(4.6,111.1)_ = 3.76, *p* < .05, *η_p_^2^* = .14]. The interaction *Block* x *Group* indicated higher EEG power modulation for Active cTBS versus Sham at block 3 (62.7 vs. 32.9%).The interaction *Electrode* x *Group* showed higher EEG synchronisation for Active cTBS compared with Sham cTBS for C3 and Cz (Fig. 3E).

### ERPow in the Low β Band

**Figure 4:**
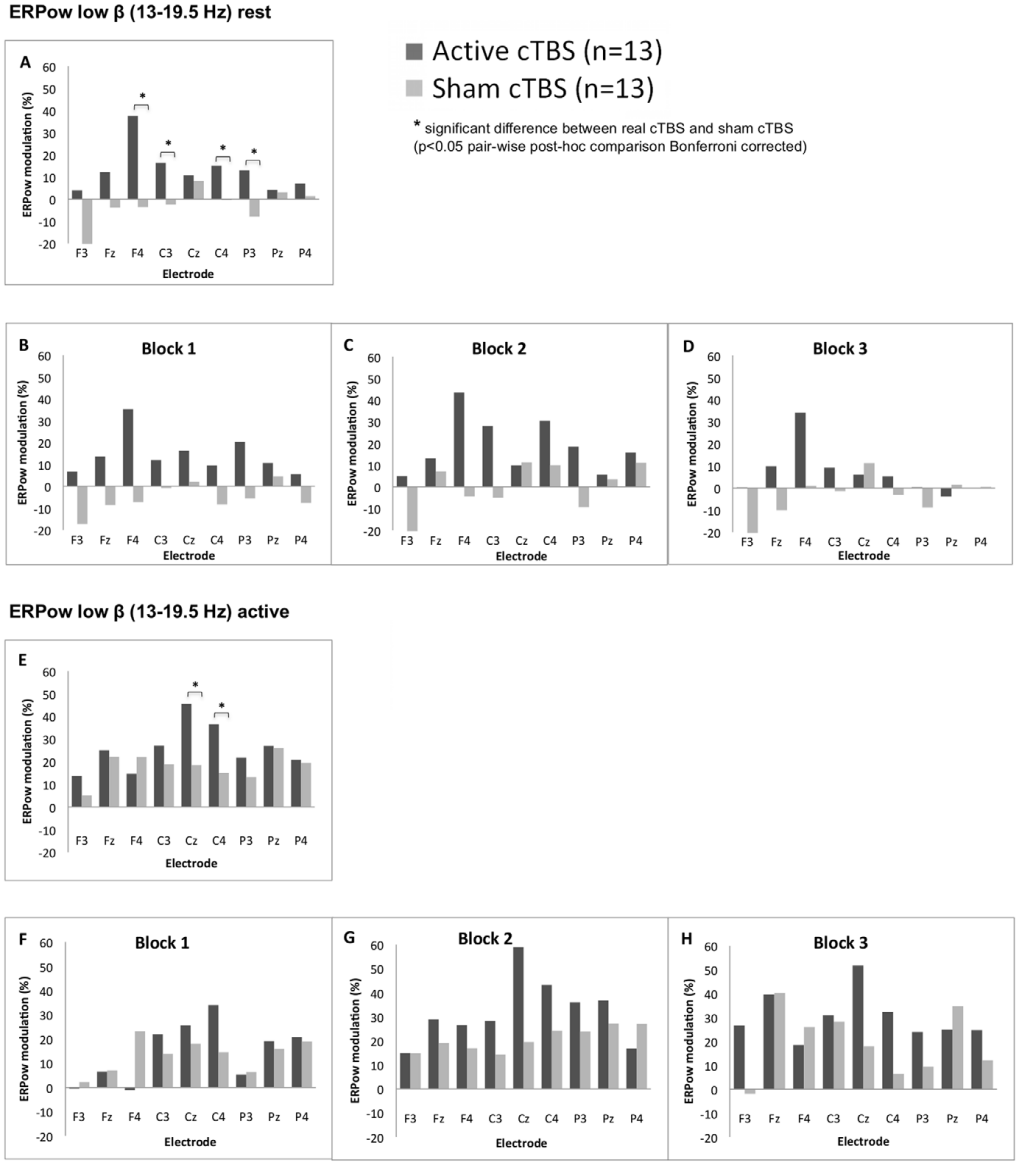
Grand average of ERPow transformation for low β (13–19.5 Hz) range. Post-cTBS after-effects are represented at rest and at active state as a function of the factors: group (Active cTBS and Sham cTBS), electrodes (F3, Fz, F4, C3, Cz, C4, P3, Pz, P4) and block of time (three levels – block one, two and three). The overall 30 minutes of post-cTBS after-effects on modulation of cortical oscillations for both groups of participants are shown at rest (label A) and active state (label E). Whereas the cTBS after-effects on ERPow separate for Block 1, Block 2 and Block 3 post stimulation are shown at rest (label B, C, and D) and at active state (label F, G, and H). Increased of neuronal synchronisation was seen for 30 minutes post-cTBS in low β band. ERPow at rest, showed higher EEG synchronisation for Active cTBS group compared with Sham cTBS group for F4, C3, C4 and P3 electrodes (See A). ERPow during active condition, showed higher synchronisation for Active cTBS compared with Sham cTBS for Cz and C4 electrodes (See E).

The ANOVA at rest for low β showed the following statistically significant main effects and interactions: *Block* [*F*_(2,48)_ = 4.52, *p* < .05, *η_p_^2^* = .16]; *Electrode* [*F*_(8,192)_ = 5.46, *p* < .001, *η_p_^2^* = .19]; *Electrode* x *Group* [*F*_(8,192)_ = 4.78, *p* < .001, *η_p_^2^* = .17]. The two-way interaction *Electrode* x *Group* showed a higher EEG power modulation for Active cTBS compared with Sham for electrode F4, C3, C4 and P3 (Fig.4A). During active condition, the ANOVAs showed statistically significant main effects and interaction: *Block* [*F*_(2,48)_ = 8.81, *p* < .01, *η_p_^2^* = .27]; *Electrode* [*F*_(5,120.8)_ = 4.34, *p* < .01, *η_p_^2^* = .15]; *Electrode* x *Group* [*F*_(5,120.8)_ = 2.92, *p* < .05, *η_p_^2^* = .11]. The two-way interaction *Electrode* x *Group* showed higher synchronisation for Active cTBS compared with Sham in Cz and C4 (Fig.4E).

### ERPow in the High β Band

**Figure 5:**
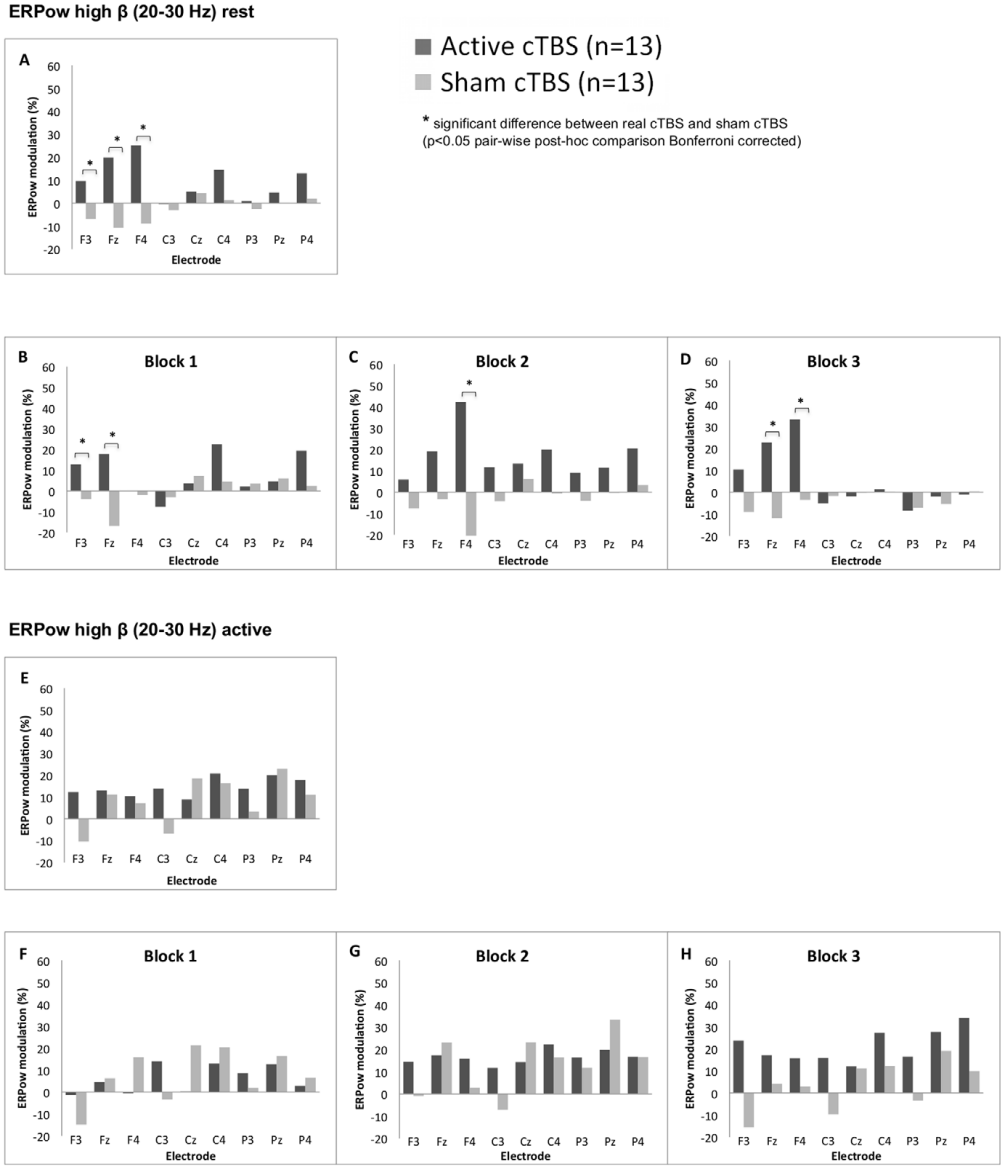
Grand average of ERPow transformation for high β (20–30.0 Hz) range. Post-cTBS after-effects are represented at rest and at active state as a function of the factors: group (Active cTBS and Sham cTBS), electrodes (F3, Fz, F4, C3, Cz, C4, P3, Pz, P4) and block of time (three levels – block one, two and three). The overall 30 minutes of post-cTBS after-effects on modulation of cortical oscillations for both groups of participants are shown at rest (label A) and active state (label E). Whereas the cTBS after-effects on ERPow separate for Block 1, Block 2 and Block 3 post stimulation are shown at rest (label B, C, and D) and at active state (label F, G, and H). Increased of neuronal synchronisation was seen for 30 minutes post-cTBS in high β band at rest for Active cTBS group compared to Sham cTBS group across the three blocks for frontal electrodes F3, Fz and F4 (See A–D).

The ANOVA of ERPow at rest for high β band did not show any significant main effects [*Block*: *F*_(2,48)_ = 2.31, *p* = .11, *η_p_^2^* = .09; *Electrode: F*_(3.7,88.7)_ = 1.36, *p* = .22, *η_p_^2^* = .05]. The significant interaction *Electrode* x *Group* [*F*_(3.7,88.7)_ = 3.67, *p* < .05, *η_p_^2^* = .13] at rest indicated higher synchronisation for Active cTBS versus Sham cTBS for frontal electrodes, F3 (9.7 vs. −6.9%), Fz (19.9 vs. −10.7%) and F4 (25.1 vs. −8.9%) (Fig.5A). The interaction *Block* x *Electrode* x *Group* [*F*_(6.7,159.7)_ = 2.59, *p* < .05, *η_p_^2^* = .1] demonstrated a higher synchronisation in Active cTBS compared to Sham across the three blocks in the frontal electrodes [block 1 of F3 (12.8 vs. −3.9%) and Fz (17.9 vs. −16.9%), block 2 of F4 (42.2 vs. −21.2%), and block 3 of Fz (22.7 vs. −11.9%) and F4 (36.3 vs. −5.6%)] (Fig.5B–D). The ANOVA of ERPow at active state for high β band showed the following statistically significant main effect and interaction [*Block*: *F*(2,48) = 3.29, *p* < .05, *η_p_^2^*  = .12; *Block* x *Group*: *F*(2,48) = 5.1, *p* < .05, *η_p_^2^* = .18]. The two-way interaction *Block* x *Group* showed a higher synchronisation for Active cTBS compared to Sham cTBS for block three (21.1 vs. 3.4%).

### MEPs

**Figure 6:**
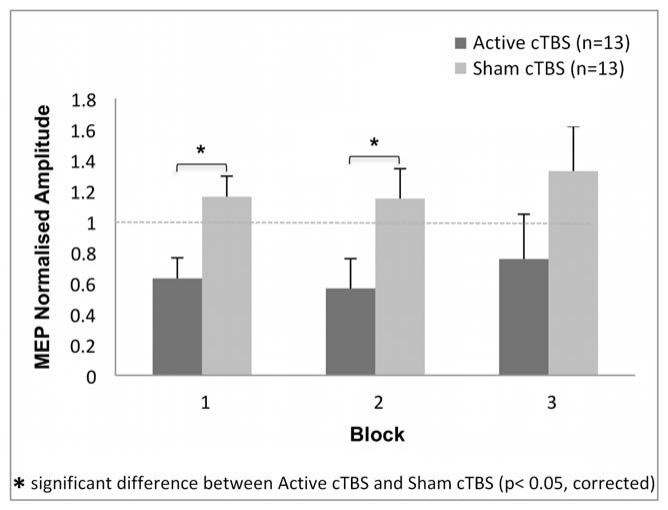
Normalised Mean Motor Evoked Potentials (MEPs) amplitude. Post-cTBS after-effects are shown at rest as a function of the factors: group (Active cTBS and Sham cTBS), and block of time (three levels – block one, two and three). Long-lasting conditioning effect of cTBS 300 pulses on MEP amplitude was seen up to 20 minutes post magnetic stimulation (Block 1 and Block 2) for Active cTBS group compared to Sham cTBS group.

Figure 6 displays the after-effects of 20 s cTBS (300 pulses) on mean normalised MEPs amplitude across blocks recorded from TE of the two groups of participants at rest. There was a significant interaction for normalised amplitude of MEPs for *Block* x *Group* [*F*_(2, 48)_ = 3.1, *p* < .05, *η_p_^2^* = .01]. Post-hoc comparisons for this significant two-ways interaction showed a significant decrease in MEPs size for Active cTBS compared to Sham cTBS in the first and second blocks [block 1 (0.63 vs. 1.16); block 2 (0.56 vs. 1.15)]. No other main factors or interactions were significant for MEPs normalised amplitude [*Latency*: *F*_(2, 48)_ = 0.17, *p* = .1, *η_p_^2^* = .007; *Latency* x *Group: F*_(2, 48)_ = 1.37, *p* = .25, *η_p_^2^* = .054].

In order to investigate the possible correlation between peripheral and cortical modulation, the Pearson coefficient was calculated between the changes in the MEP amplitude and the ERPow values in the three blocks of the Active cTBS session for all frequency bands over the C3 electrode. C3 was selected due to its location to the motor cortex. No significant correlations were found between the decrease of the MEPs amplitude and the increase of the EEG synchronisation over the C3 electrodes for all the frequency bands analysed in all the three blocks of time. [θ block 1 (*r* = −.19, *p* = .52), block 2 (*r* = .30, *p* = .31), block 3 (*r* = .06, *p* = .84)]; [low α block 1 (*r* = .04, *p* = .91), block 2 (*r* = −.16, *p* = .61), block 3 (*r* = .07, *p* = .81)]; [µ block 1 (*r* = −.40, *p* = .18), block 2 (*r* = .03, *p* = .93), block 3 (*r* = .16, *p* = .61)]; [low β block 1 (*r* = .01, *p* = .99), block 2 (*r* = .06, *p* = .74), block 3 (*r* = .36, *p* = .23)]; [high β block 1 (*r* = .29, *p* = .33), block 2 (*r* = −.23, *p* = .45), block 3 (*r* = .21, *p* = .49)].

### RT Task

**Figure 7:**
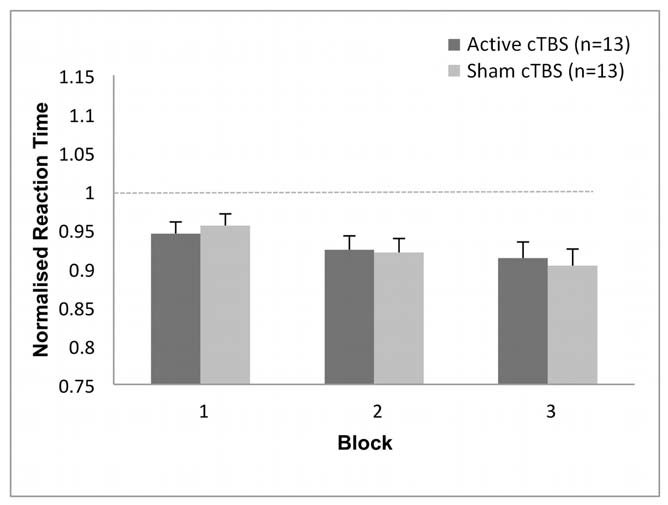
Normalised Mean Response Times. Post-cTBS after-effects were assessed at active state, during the execution of a Reaction Time (RT) task. Absence of long-lasting conditioning effect of cTBS 300 pulses on RT task performance for Active cTBS group compared to Sham cTBS group, showing a practice effect.

Figure 7 illustrates the normalised RT across blocks and between the two groups of participants. The only significant main effect for normalised RT was *block* [*F*_(2, 48)_  =  7.7, *p* < .005, *η_p_^2^* =  .24]. Post-hoc comparisons for *block* showed for all participants a significant decrease in RT between block 1 and both blocks 2 and 3 (0.95 vs.0.92, 0.91). No other main factors or interactions were significant for normalised RT and accuracy.

## Discussion

The aim of this study was to compare the temporal dynamics of cortical excitability using behavioural and electrophysiological measurements post-cTBS. The other goal was to examine how preconditioning motor cortex with high-frequency cTBS affected the subsequent patterns of cortical synchronisation, the mechanism believed to be important for cortical information processing [29], [33]. We quantified the modulation of behavioural measures (MEPs and RT) and EEG oscillations (ERPow) at both rest and active state, during the execution of a choice RT task, post-cTBS. By examining the mean normalised EEG responses to high-frequency rTMS in a 30-min window after stimulation, our main finding was a longer increase of neuronal synchronisation (at least 30-min) compared with relatively shorter (20-min) suppression of MEPs amplitude. This finding showed that besides MEPs, EEG could be another sensitive tool to measure index of plasticity after theta burst magnetic stimulation on a large neuronal network. We also observed β as the predominant frequency band modulated by low-intensity cTBS. Since β is mostly represented at cortical level during awake and alert states of the brain, our finding supported the hypothesis that TBS acts more on cortical level rather than deeper structures. The θ oscillation was also seen post-cTBS at rest, and these generations of θ oscillation could be due to the presence of independent cortical θ generators over the motor network. However, there was no significant modulation of RT induced by cTBS protocol. Although enhance synchronisation may interfere with cortical information processing, our result suggests that a healthy motor system is able to compensate for transient perturbation by cTBS.

### cTBS Effects on Oscillatory Neural Activity

EEG data was analysed using spectral analysis of event-related power to assess the local synchronisation of the neuronal oscillatory activity. Cranio-cerebral projections were used to characterise the relationship between standard 10–20 positions and the anatomical localisation underlying cortical structures [25], [34]. F3 and F4 electrodes are usually located above the left and right frontal lobe middle frontal gyrus, C3–C4 electrodes lie over the left and right parietal lobe post central gyrus, and P3–P4 electrodes lie over left and right parietal lobe angular gyrus.

In this study, we demonstrated that theta burst magnetic stimulation induced a general increase in synchrony of the underlying neuronal populations of different cortical regions across different frequency bands, with a 20-min suppression of MEPs. The increase in ERPow was observed in the three blocks, for at least 30-min after the conditioning train of stimulation, showing a longer lifetime of TBS-induced EEG changes than on the behavioural measurement. Moreover a negative result was the lack of correlation of MEPs size on modulation of cortical oscillations after cTBS. A possible explanation for the discrepancy of behavioural and electrophysiological findings in our study was that cTBS works on MEPs and EEG oscillatory activities through different mechanisms [20]; MEP amplitudes reflected the neuronal excitability of the target muscle and affected by spinal excitability whereas EEG oscillations were the sum activity of a large population of cortical neurons [35]. Another explanation could be the sensitivity of EEG measurement compared with MEPs to investigate LTP and LTD-like mechanisms induced by rTMS [18], [23]. Since LTP and LTD of synaptic plasticity are monosynaptic events, it would thus be advantageous to monitor cortical readout that is linked by a single synapse to the TMS pulse [18]. However, MEP that is commonly used in rTMS experiments as indicator for cortical excitability is a polysynaptic event separated by at least three synapses (synapses onto corticospinal neurons, synapses onto motor neurons of the spinal cord, and the neuromuscular synapses) from the TMS source [18], [19]. Instead, EEG signal is linked by a single synapse of synchronous excitatory and inhibitory input of pyramidal dendrites to TMS[36], therefore probably able to provide a more accurate interpretation of cortical conditioning of high-frequency rTMS. The absence of correlation between the modulation of MEPs size and cortical oscillations after cTBS could also simply be explained by the high inter-trial variability in MEP size. For each participant, only ten single TMS pulses have been used to define the MEP measure in each of the four blocks of the entire experimental session. This small number of repetitions for MEPs was probably not enough to obtain a totally reliable measure.

Our study revealed β as the most dominant frequency band modulated post-cTBS both at rest and active states. Physiologically, β oscillations were associated with motor activity and are cortically generated [37]. This finding supported the hypothesis that TBS would mainly involve modulation of cerebral cortex rather than deeper structures [6], [14]. During TBS, subthreshold stimulus intensity is used (80% AMT) and this stimulus is insufficient for the activation of the descending pathways [38]. Study revealed that the higher frequency brain rhythms of β and γ have the most dominant effect on the stimulated hemisphere post-cTBS [7]. In our study, we demonstrated the increase synchronisation of high β mainly in the frontal area for both left and right frontal lobe middle frontal gyrus. It was interesting that the most pronounced and persistent effect on the stimulated hemisphere was on the fast activity (high β), which has been proposed as the main neurophysiological correlate of rapid information processing [39]. Since dynamic balance of synchronisation and desynchronisation has been implicated as a possible mechanism of cortical information processing [33], thus cTBS might impair cortical information coding through enhanced synchronisation of high-frequency brain rhythms [7].

EEG analysis during resting state post-cTBS also revealed increase EEG synchronisation for θ band. This supported our previous study that showed a higher cortical oscillations of θ (4–7 Hz) compared to µ (10–12 Hz) and β (13–30 Hz) up to 20 seconds after intermittent trains of 60 pulses of high frequency magnetic stimulation (∼ 11 Hz) to the primary motor cortex at rest [26]. We proposed that the increase in θ power modulation may be due to the independent theta generators that are not confined to the hippocampus but also present near the brain surface as demonstrated by animal studies [40], [41], and human intracranial EEG recordings [41]–[44]. In the present study, we observed the global topography of θ changes in ERPow at rest involving electrodes from frontal lobe middle frontal gyrus, parietal lobe post central gyrus and parietal lobe angular gyrus, thus supporting the involvement of widespread cortical θ generators over the motor network after cTBS. However, we should be cautious in our interpretation because in our study, we measured cortical oscillations in healthy humans using surface EEG. A study using invasive intracranial electrodes on patients will give a more accurate interpretation than surface EEG.

Our result of high α band in the active state showed a modulation with an increase of ERPow in C3 and Cz electrodes. This distinct high alpha frequency band has been called µ rhythm when it is present over central Rolandic cortical areas [45]. The increase µ synchronisation in the active state rather than at rest is rather surprising, because µ rhythm is more dominant during quiet wakefulness and is blocked by motor movements or somatosensory stimuli [46]. Although previously µ rhythm was thought as merely epiphenomena without functional significance, recent research reveals that µ is more than an idling state of sensorimotor cortex and instead reflects integrative sensory and motor processes with important information processing function [47], [48].

### cTBS Effects on MEPs

The excitability of the corticospinal system before and after Active and Sham cTBS was measured using single-pulse TMS to evoke EMG responses (MEPs) from right hand muscle for both groups of participants at rest. Figure 6 shows 20-min long-lasting suppression on MEPs after 300 pulses of high-frequency TMS for Active cTBS only. Our findings replicated several studies, which demonstrated how a low-intensity conditioning train of stimulation suppresses cortical excitability, emulating LTD events [16], [38], [49], [50].

At present, the reasons for the suppressive effects of cTBS on the size of MEPs remain debatable. Some authors proposed the involvement of N-methyl-D-aspartate receptors (NMDA-r) as demonstrated by pharmacological studies [14], [51]–[54]. Other researchers highlighted the role of inhibitory cortical systems mainly through γ-aminobutyric acid receptor (GABA-r) modulation [55], [56]. Alternative explanations included TBS effects on gene expression and protein levels [57]. The experiments with single trains of cTBS in humans suggested that inhibition was built up slower but saturated later than facilitation, hence the suppressive effect of MEPs after longer lasting cTBS [2].

### cTBS Effects on RT Task Performance

Overall, the two groups of participants had improved performance in terms of shorter RT across blocks of time. This suggests a possible practice effect, where mean RT invariably decreases when subjects perform the same cognitive task repeatedly [58]. The occurrence of the practice effect may involve the adjustments of response strategy [58].

Our study showed that motor latency responses on correct trials had no significant interactions across blocks for either Active cTBS or Sham cTBS. This result contradicted our earlier hypothesis that cTBS would increase RT in the contralateral (right) hand to the site of stimulation. Previous studies demonstrated that conditioning the left motor cortex with cTBS produced clear increase in simple RT in the right conditioned hand up to 10-min after the train of magnetic stimulation and a decrease in RT in the left unconditioned hand 30-min after cTBS [59]. In another study, response latency in a choice RT task was delayed in both hands with cTBS applied over either left or right dorsal premotor cortex (PMd) suggesting that TBS leads to widespread long-terms forms of interference and complex effects on behaviour [60]. In our study, the absence of an effect on choice RT post-cTBS could be due to the fact that we perturbed the activity of the primary motor cortex, which is more involved in the motor execution of a simple RT paradigm. We did not directly interfere with the processing of the premotor cortex, which is more involved in motor preparation during a choice RT [61]. Several studies have shown MEP suppression after magnetic stimulation but without any effect on simple RT performances [62], [63]. The fact that the movement was not compromised in their studies and ours, indicated that a healthy motor system was able to functionally compensate, to some extent, for temporary deficits induced by TBS perturbation of cortical excitability [62], [63].

## Conclusions

Overall our present work demonstrated that the cTBS was associated with increase neuronal synchronisation as assessed by surface EEG. This suggests a probable link between network oscillations and plasticity-like mechanisms after theta burst stimulation. Although, it was tempting to associate increase neuronal synchronisation with mechanism of LTD, the limitation of inferences of EEG on micro-level make us cautious to do so. Surface EEG will only record neural activity if there is synchronicity on a large scale underlying the electrode. Therefore, our result can only be interpreted on a macroscopic scale but not on a micro-level, which cannot be computed with scalp EEG. Nevertheless, due to the rise of therapeutic protocol using rTMS, it is important to extend previous research using EEG to probe treatment efficacy and the mechanisms of synaptic plasticity post rTMS [21], [64]. Future studies of combined TBS/EEG should investigate the time course of cortical oscillations by cTBS beyond the 30-minutes temporal window to further examine the time course of modulation of cortical oscillations of this brain stimulation protocol.

## References

[1] Hallett M (2007). Transcranial magnetic stimulation: a primer.. Neuron.

[2] Huang YZ, Edwards MJ, Rounis E, Bhatia KP, Rothwell JC (2005). Theta burst stimulation of the human motor cortex.. Neuron.

[3] Paulus W (2005). Toward establishing a therapeutic window for rTMS by theta burst stimulation.. Neuron.

[4] Grossheinrich N, Rau A, Pogarell O, Hennig-Fast K, Reinl M (2009). Theta burst stimulation of the prefrontal cortex: safety and impact on cognition, mood, and resting electroencephalogram.. Biol Psychiatry.

[5] Rossi S, Hallett M, Rossini PM, Pascual-Leone A (2009). Safety, ethical considerations, and application guidelines for the use of transcranial magnetic stimulation in clinical practice and research.. Clin Neurophysiol.

[6] Cardenas-Morales L, Nowak DA, Kammer T, Wolf RC, Schonfeldt-Lecuona C (2010). Mechanisms and applications of theta-burst rTMS on the human motor cortex.. Brain Topogr.

[7] Schindler K, Nyffeler T, Wiest R, Hauf M, Mathis J (2008). Theta burst transcranial magnetic stimulation is associated with increased EEG synchronization in the stimulated relative to unstimulated cerebral hemisphere.. Neurosci Lett.

[8] Llinas RR, Ribary U, Jeanmonod D, Kronberg E, Mitra PP (1999). Thalamocortical dysrhythmia: A neurological and neuropsychiatric syndrome characterized by magnetoencephalography.. Proc Natl Acad Sci U S A.

[9] Jeanmonod D, Schulman J, Ramirez R, Cancro R, Lanz M (2003). Neuropsychiatric thalamocortical dysrhythmia: surgical implications.. Neurosurgery clinics of North America.

[10] Llinas R, Urbano FJ, Leznik E, Ramirez RR, van Marle HJ (2005). Rhythmic and dysrhythmic thalamocortical dynamics: GABA systems and the edge effect.. Trends Neurosci.

[11] Schulman JJ, Cancro R, Lowe S, Lu F, Walton KD (2011). Imaging of thalamocortical dysrhythmia in neuropsychiatry.. Frontiers in human neuroscience.

[12] Ziemann U (2011). Transcranial magnetic stimulation at the interface with other techniques: a powerful tool for studying the human cortex.. Neuroscientist.

[13] McAllister SM, Rothwell JC, Ridding MC (2011). Cortical oscillatory activity and the induction of plasticity in the human motor cortex.. Eur J Neurosci.

[14] Huang YZ, Chen RS, Rothwell JC, Wen HY (2007). The after-effect of human theta burst stimulation is NMDA receptor dependent.. Clin Neurophysiol.

[15] Nyffeler T, Wurtz P, Luscher HR, Hess CW, Senn W (2006). Repetitive TMS over the human oculomotor cortex: comparison of 1-Hz and theta burst stimulation.. Neurosci Lett.

[16] Stefan K, Gentner R, Zeller D, Dang S, Classen J (2008). Theta-burst stimulation: remote physiological and local behavioral after-effects.. Neuroimage.

[17] Suppa A, Ortu E, Zafar N, Deriu F, Paulus W (2008). Theta burst stimulation induces after-effects on contralateral primary motor cortex excitability in humans.. J Physiol.

[18] Huerta PT, Volpe BT (2009). Transcranial magnetic stimulation, synaptic plasticity and network oscillations.. J Neuroeng Rehabil.

[19] Siebner HR, Rothwell J (2003). Transcranial magnetic stimulation: new insights into representational cortical plasticity.. Exp Brain Res.

[20] Maki H, Ilmoniemi RJ (2010). EEG oscillations and magnetically evoked motor potentials reflect motor system excitability in overlapping neuronal populations.. Clin Neurophysiol.

[21] Thut G, Miniussi C (2009). New insights into rhythmic brain activity from TMS-EEG studies.. Trends Cogn Sci.

[22] Thut G, Pascual-Leone A (2010). Integrating TMS with EEG: How and what for?. Brain Topogr.

[23] Thut G, Pascual-Leone A (2010). A review of combined TMS-EEG studies to characterize lasting effects of repetitive TMS and assess their usefulness in cognitive and clinical neuroscience.. Brain Topogr.

[24] Oldfield RC (1971). The assessment and analysis of handedness: the Edinburgh inventory.. Neuropsychologia.

[25] Jurcak V, Tsuzuki D, Dan I (2007). 10/20, 10/10, and 10/5 systems revisited: their validity as relative head-surface-based positioning systems.. Neuroimage.

[26] Azila Noh N, Fuggetta G (2011). Human cortical theta reactivity to high-frequency repetitive transcranial magnetic stimulation..

[27] Formaggio E, Storti SF, Avesani M, Cerini R, Milanese F (2008). EEG and FMRI coregistration to investigate the cortical oscillatory activities during finger movement.. Brain Topogr.

[28] Fuggetta G, Pavone EF, Fiaschi A, Manganotti P (2008). Acute modulation of cortical oscillatory activities during short trains of high-frequency repetitive transcranial magnetic stimulation of the human motor cortex: a combined EEG and TMS study.. Hum Brain Mapp.

[29] Sauseng P, Klimesch W (2008). What does phase information of oscillatory brain activity tell us about cognitive processes?. Neurosci Biobehav Rev.

[30] Brignani D, Manganotti P, Rossini PM, Miniussi C (2008). Modulation of cortical oscillatory activity during transcranial magnetic stimulation.. Human brain mapping.

[31] Veniero D, Brignani D, Thut G, Miniussi C (2011). Alpha-generation as basic response-signature to transcranial magnetic stimulation (TMS) targeting the human resting motor cortex: A TMS/EEG co-registration study.. Psychophysiology.

[32] Pfurtscheller G, Lopes da Silva FH (1999). Event-related EEG/MEG synchronization and desynchronization: basic principles.. Clinical neurophysiology : official journal of the International Federation of Clinical Neurophysiology.

[33] Pareti G, De Palma A (2004). Does the brain oscillate? The dispute on neuronal synchronization.. Neurol Sci.

[34] Okamoto M, Dan H, Sakamoto K, Takeo K, Shimizu K (2004). Three-dimensional probabilistic anatomical cranio-cerebral correlation via the international 10–20 system oriented for transcranial functional brain mapping.. Neuroimage.

[35] Komssi S, Kahkonen S (2006). The novelty value of the combined use of electroencephalography and transcranial magnetic stimulation for neuroscience research.. Brain Res Rev.

[36] Taylor PC, Walsh V, Eimer M (2008). Combining TMS and EEG to study cognitive function and cortico-cortico interactions.. Behav Brain Res.

[37] Salenius S, Hari R (2003). Synchronous cortical oscillatory activity during motor action.. Curr Opin Neurobiol.

[38] Di Lazzaro V, Pilato F, Saturno E, Oliviero A, Dileone M (2005). Theta-burst repetitive transcranial magnetic stimulation suppresses specific excitatory circuits in the human motor cortex.. J Physiol.

[39] Rosanova M, Casali A, Bellina V, Resta F, Mariotti M (2009). Natural frequencies of human corticothalamic circuits.. J Neurosci.

[40] Leung LW, Borst JG (1987). Electrical activity of the cingulate cortex. I. Generating mechanisms and relations to behavior.. Brain Res.

[41] Silva LR, Amitai Y, Connors BW (1991). Intrinsic oscillations of neocortex generated by layer 5 pyramidal neurons.. Science.

[42] Caplan JB, Madsen JR, Raghavachari S, Kahana MJ (2001). Distinct patterns of brain oscillations underlie two basic parameters of human maze learning.. J Neurophysiol.

[43] Kahana MJ, Seelig D, Madsen JR (2001). Theta returns.. Curr Opin Neurobiol.

[44] Raghavachari S, Lisman JE, Tully M, Madsen JR, Bromfield EB (2006). Theta oscillations in human cortex during a working-memory task: evidence for local generators.. J Neurophysiol.

[45] Pfurtscheller G, Brunner C, Schlogl A, Lopes da Silva FH (2006). Mu rhythm (de)synchronization and EEG single-trial classification of different motor imagery tasks.. Neuroimage.

[46] Makeig S, Westerfield M, Jung TP, Enghoff S, Townsend J (2002). Dynamic brain sources of visual evoked responses.. Science.

[47] Niedermeyer E, Goldszmidt A, Ryan D (2004). “Mu rhythm status” and clinical correlates.. Clin EEG Neurosci.

[48] Pineda JA (2005). The functional significance of mu rhythms: translating “seeing” and “hearing” into “doing”.. Brain Res Brain Res Rev.

[49] Hernandez RV, Navarro MM, Rodriguez WA, Martinez JL, LeBaron RG (2005). Differences in the magnitude of long-term potentiation produced by theta burst and high frequency stimulation protocols matched in stimulus number.. Brain Res Brain Res Protoc.

[50] Zhang Y, Llinas RR, Lisman JE (2009). Inhibition of NMDARs in the Nucleus Reticularis of the Thalamus Produces Delta Frequency Bursting.. Front Neural Circuits.

[51] Di Lazzaro V, Pilato F, Dileone M, Profice P, Oliviero A (2008). The physiological basis of the effects of intermittent theta burst stimulation of the human motor cortex.. J Physiol.

[52] Huang CC, Hsu KS (2008). The role of NMDA receptors in regulating group II metabotropic glutamate receptor-mediated long-term depression in rat medial prefrontal cortex.. Neuropharmacology.

[53] Huang YZ, Rothwell JC, Edwards MJ, Chen RS (2008). Effect of physiological activity on an NMDA-dependent form of cortical plasticity in human.. Cereb Cortex.

[54] Ridding MC, Rothwell JC (2007). Is there a future for therapeutic use of transcranial magnetic stimulation?. Nat Rev Neurosci.

[55] Thickbroom GW (2007). Transcranial magnetic stimulation and synaptic plasticity: experimental framework and human models.. Exp Brain Res.

[56] Trippe J, Mix A, Aydin-Abidin S, Funke K, Benali A (2009). Theta burst and conventional low-frequency rTMS differentially affect GABAergic neurotransmission in the rat cortex.. Exp Brain Res.

[57] Aydin-Abidin S, Trippe J, Funke K, Eysel UT, Benali A (2008). High- and low-frequency repetitive transcranial magnetic stimulation differentially activates c-Fos and zif268 protein expression in the rat brain.. Exp Brain Res.

[58] Dutilh G, Vandekerckhove J, Tuerlinckx F, Wagenmakers EJ (2009). A diffusion model decomposition of the practice effect.. Psychon Bull Rev.

[59] Mochizuki H, Franca M, Huang YZ, Rothwell JC (2005). The role of dorsal premotor area in reaction task: comparing the “virtual lesion” effect of paired pulse or theta burst transcranial magnetic stimulation.. Exp Brain Res.

[60] Mochizuki H, Furubayashi T, Hanajima R, Terao Y, Mizuno Y (2007). Hemoglobin concentration changes in the contralateral hemisphere during and after theta burst stimulation of the human sensorimotor cortices.. Exp Brain Res.

[61] Perfetti B, Moisello C, Landsness EC, Kvint S, Pruski A (2011). Temporal evolution of oscillatory activity predicts performance in a choice-reaction time reaching task.. J Neurophysiol.

[62] Iyer MB, Schleper N, Wassermann EM (2003). Priming stimulation enhances the depressant effect of low-frequency repetitive transcranial magnetic stimulation.. J Neurosci.

[63] Stinear CM, Barber PA, Coxon JP, Verryt TS, Acharya PP (2009). Repetitive stimulation of premotor cortex affects primary motor cortex excitability and movement preparation.. Brain Stimul.

[64] Miniussi C, Thut G (2010). Combining TMS and EEG offers new prospects in cognitive neuroscience.. Brain Topogr.

